# Magnetic chitosan stabilized Cu(II)-tetrazole complex: an effective nanocatalyst for the synthesis of 3-imino-2-phenylisoindolin-1-one derivatives under ultrasound irradiation

**DOI:** 10.1038/s41598-022-10591-4

**Published:** 2022-04-25

**Authors:** Mahmoud Nasrollahzadeh, Nasrin Shafiei, Yasin Orooji

**Affiliations:** 1 College of Geography and Environmental Sciences, Zhejiang Normal University, Jinhua, 321004 People’s Republic of China; 2 Department of Chemistry, Faculty of Science, University of Qom, 37185-359 Qom, Iran; 3 Co-Innovation Center of Efficient Processing and Utilization of Forest Resources, College of Materials Science and Engineering, Nanjing Forestry University, Nanjing, 210037 People’s Republic of China; 4 State Key Laboratory of Materials-Oriented Chemical Engineering, College of Chemical Engineering, Nanjing Tech University, Nanjing, 211816 People’s Republic of China

**Keywords:** Carbohydrates, Metals

## Abstract

In the present research, a recyclable catalyst has been prepared via a simple approach using chitosan as a linear polysaccharide. This paper reports the synthesis of novel copper(II) complex of 5-phenyl-1*H*-tetrazole immobilized on magnetic chitosan (MCS@PhTet@Cu(II)) as an effective catalyst. Transmission electron microscopy (TEM), field emission scanning electron microscopy (FESEM), vibrating sample magnetometer (VSM), Fourier-transform infrared spectroscopy (FT-IR), X-ray diffraction (XRD), energy-dispersive X-ray spectroscopy (EDS), and inductively coupled plasma mass spectrometry (ICP-MS) techniques were applied for the characterization of the catalyst. The catalytic activity of MCS@PhTet@Cu(II) was evaluated in the ultrasound-assisted synthesis of 3-imino-2-phenylisoindolin-1-one derivatives via the reaction between benzoyl chloride and arylcyanamides in ethanol at ambient temperature. Utilizing a wide variety of arylcyanamides under mild conditions, no use of toxic organic solvents, moderate reaction time, high yields along with catalyst excellent reusability and easy separation of the products without any tedious separation techniques, made this method a novel and simple process. The resulting heterogeneous catalyst showed valuable advantages such as easier work-up, better stability, and greater separation ability using an external magnet. The catalyst showed high efficacy and recyclability even after five cycles with no significant loss of its efficacy. The present methodology provides a path for the preparation of structurally diverse heterocyclic compounds, which may exhibit important biological activity.

## Introduction

An important class of organic reactions is cycloaddition reactions. These reactions afford medicinally and biologically valuable heterocyclic compounds which include one or more heteroatoms, especially O or N^1–9^. Many natural products including alkaloids, vitamins, and hormones contain nitrogen-based five-membered heterocycles. In addition to their vitality to living organisms, they are also industrially significant for the preparation of pharmaceuticals, dyes, pesticides, herbicides, etc.^10–13^. Among heterocycles, isoindolines and their derivatives are known as an attractive group of five-membered *N*-based heterocycles in organic, coordination and medicinal chemistry^14–16^. Various substrates and different methods have been reported to synthesize isoindoline derivatives^17–20^.

Cyanamide is present in many bioactive molecules. It has a unique structure with exceptional properties making it appropriate as a building block in organic synthesis^6,21–25^. Cyanamides contain a nucleophilic "amino" center and an electrophilic "cyano" part, making them fascinating compounds for different reactions such as addition, cycloaddition, and cyclotrimerization^26,27^.

Generally, most organic reactions take place using a catalyst or ultrasound (US) irradiation as a driving force^28,29^. US presents high pressures and energies at a short time and the catalyst activates a special region of the molecule to proceed with the reaction.

Among various catalysts, heterogeneous ones are of significant interest since they are non-toxic, cost-effective, and efficient^6,30–42^. Heterogeneous catalysts do not dissolve in the reaction media and hence are easier to recover from the mixture by different methods such as filtration, centrifugation, separation by an external magnet, etc. They are also reusable and maintain their efficacy even after several runs^43–51^. Among them, magnetite nanoparticles have large surface areas, small sizes, and present magnetic properties to the catalyst^47–50^. Magnetic heterogeneous catalysts are easy to prepare, cost-effective, stable, biocompatible, and easily separated by a magnetic field^6,47–50^.

**Figure 1:**
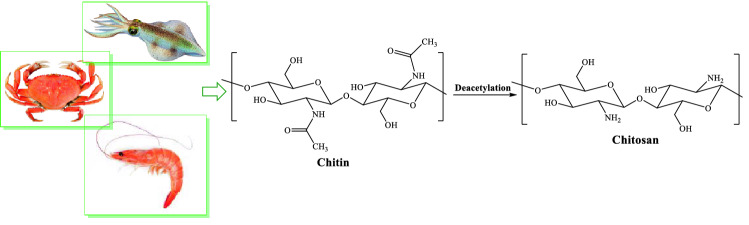
Natural sources of chitosan.

Today, biopolymers are used in the different fields^6,47,52–56^. Among biopolymers, chitosan is an *N*-deacetylated type of chitin, found in nature, such as in Crabs, Shrimp, squid, etc. (Fig. 1). Chitosan (CS) is an abundant, biodegradable, hydrophilic, and natural polysaccharide, which affords desirable structural features for mechanical and chemical modifications^6,47,52^. It is an ideal support for the immobilization of metal nanoparticles due to its high stability, high surface area and low cost. Functionalization of chitosan biopolymer with Fe_3_O_4_ affords magnetic property, which causes separation from the reaction media^6,47^.

In addition, the application of tetrazoles as efficient ligands in the catalysis has recently drawn great attention. Tetrazoles are cyclic compounds with four nitrogen atoms and a good ability to coordinate metal particles. These molecules and their anions (tetrazolates) can act as multi-dentate ligands with different coordination modes to prepare efficient catalysts through complexation with metal ions^6,48^.

**Scheme 1:**
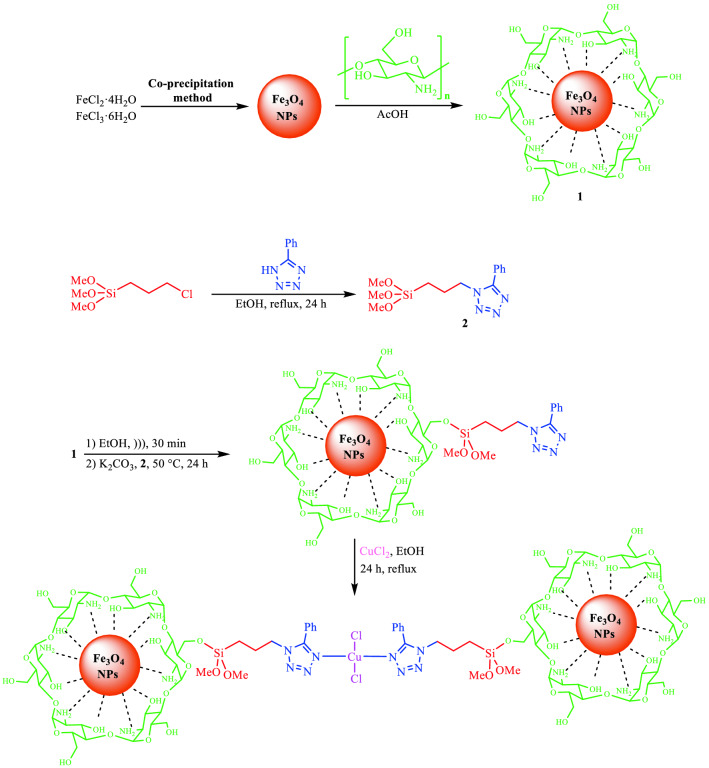
Preparation of MCS@PhTet@Cu(II).

**Scheme 2:**
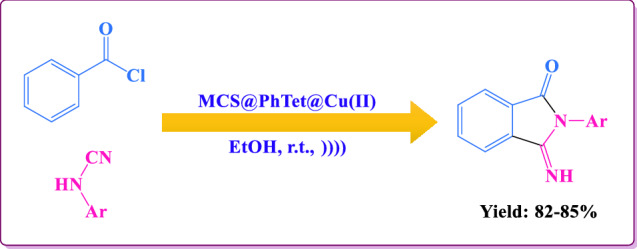
The general reaction between benzoyl chloride and arylcyanamides using the catalyst under ultrasound irradiation.

In this study, a novel catalytic system based on magnetic chitosan and 5-phenyl-1*H*-tetrazole has been designed and prepared (Scheme 1). The prepared catalyst has then been used to synthesize 3-imino-2-phenylisoindolin-1-one derivatives by the reaction between benzoyl chloride and arylcyanamides in ethanol green solvent at ambient temperature (Scheme 2). Our proposed catalyst is efficient, non-toxic, magnetically recoverable from the reaction media, and reusable for five times with no noteworthy decay of activity.

## Results and discussion

Synthesis of MCS@PhTet@Cu(II) was carried out by a simple technique, as shown in Scheme 1. Firstly, Fe_3_O_4_ nanoparticles were prepared via the co-precipitation method. The synthesized Fe_3_O_4_ nanoparticles (NPs) were then added to the chitosan solution at room temperature to form Fe_3_O_4_-chitosan (MCS). Next, 5-amino-1*H*-tetrazole and (3-chloropropyl)trimethoxysilane were mixed in a flask and the mixture obtained was refluxed for 24 h in EtOH. In the next step, the dispersed chitosan solution in ethanol and K_2_CO_3_ base were added to the solution prepared in the previous step and the mixture was stirred at 50 °C for 24 h to yield magnetic chitosan@5-phenyl-1*H*-tetrazole (MCS@PhTet). Finally, MCS@PhTet and CuCl_2_ were mixed in EtOH and heated at reflux for 24 h. The obtained catalyst was then collected using an external magnet, washed with EtOH, dried, and used as an effective catalyst in the reaction of benzoyl chloride with arylcyanamides. The prepared catalyst performed well in the synthesis of 3-imino-2-phenylisoindolin-1-one derivatives and afforded the products in high yields. The products were isolated without the use of any chromatographic techniques.

### Characterization of MCS@PhTet@Cu(II)

The structure and morphology of MCS@PhTet@Cu(II) were characterized by FT-IR, XRD, FESEM, EDS, TEM, ICP-MS, and VSM.

**Figure 2:**
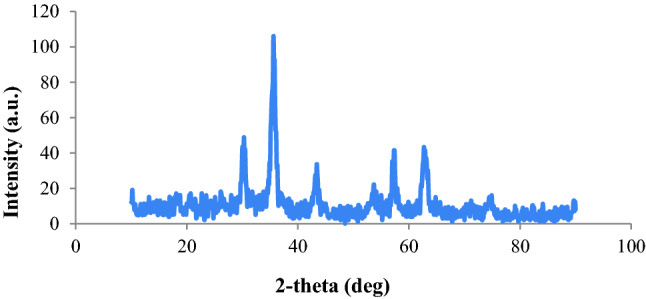
XRD analysis of MCS@PhTet@Cu(II).

XRD analysis was employed to characterize the crystalline structure of the catalyst (Fig. 2). The peaks at 2θ = 30.2° (2 2 0), 35.8° (3 1 1), 62.8° (5 3 3), 57.2° (4 4 0), 53.7° (5 1 1), and 43.5° (4 0 0) refer to the cubic structure of Fe_3_O_4_, indicating the crystalline structure of Fe_3_O_4_. Furthermore, the peaks at 2θ = 74.5°, 50.8°, and 43.5° confirm the presence of Cu in the catalyst.

**Figure 3:**
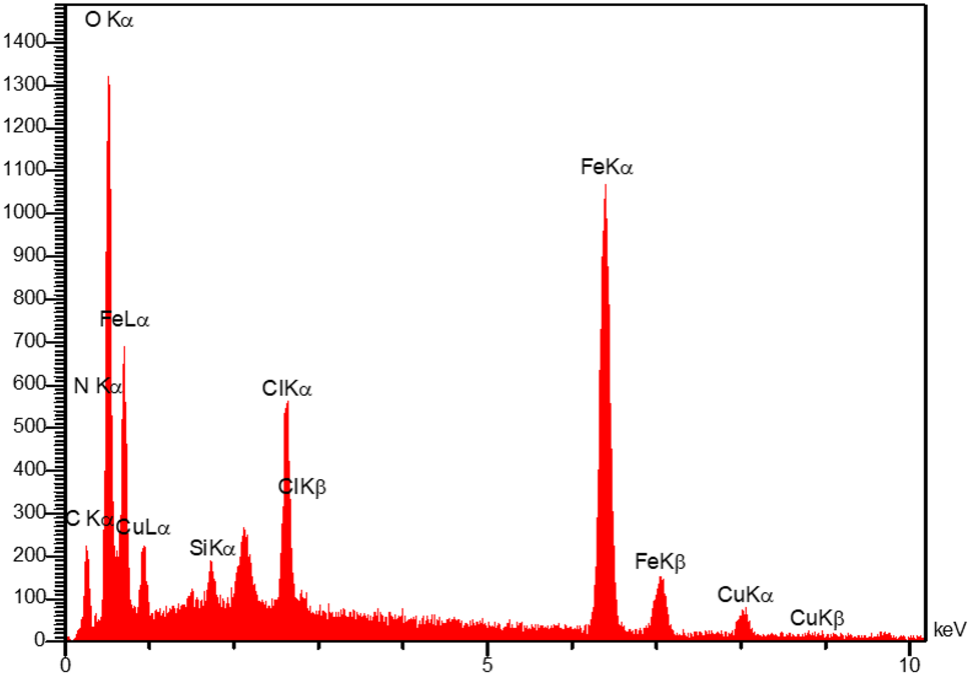
EDS spectrum of MCS@PhTet@Cu(II).

The EDS spectrum of the catalyst was used to determine the elements of the catalyst. The EDS spectrum in Fig. 3 demonstrates the presence of Fe, Cu, O, and Si in MCS@PhTet@Cu(II). Moreover, the exact amount of Cu in MCS@PhTet@Cu(II) obtained by ICP-MS technique was 4.1 wt.%.

**Figure 4:**
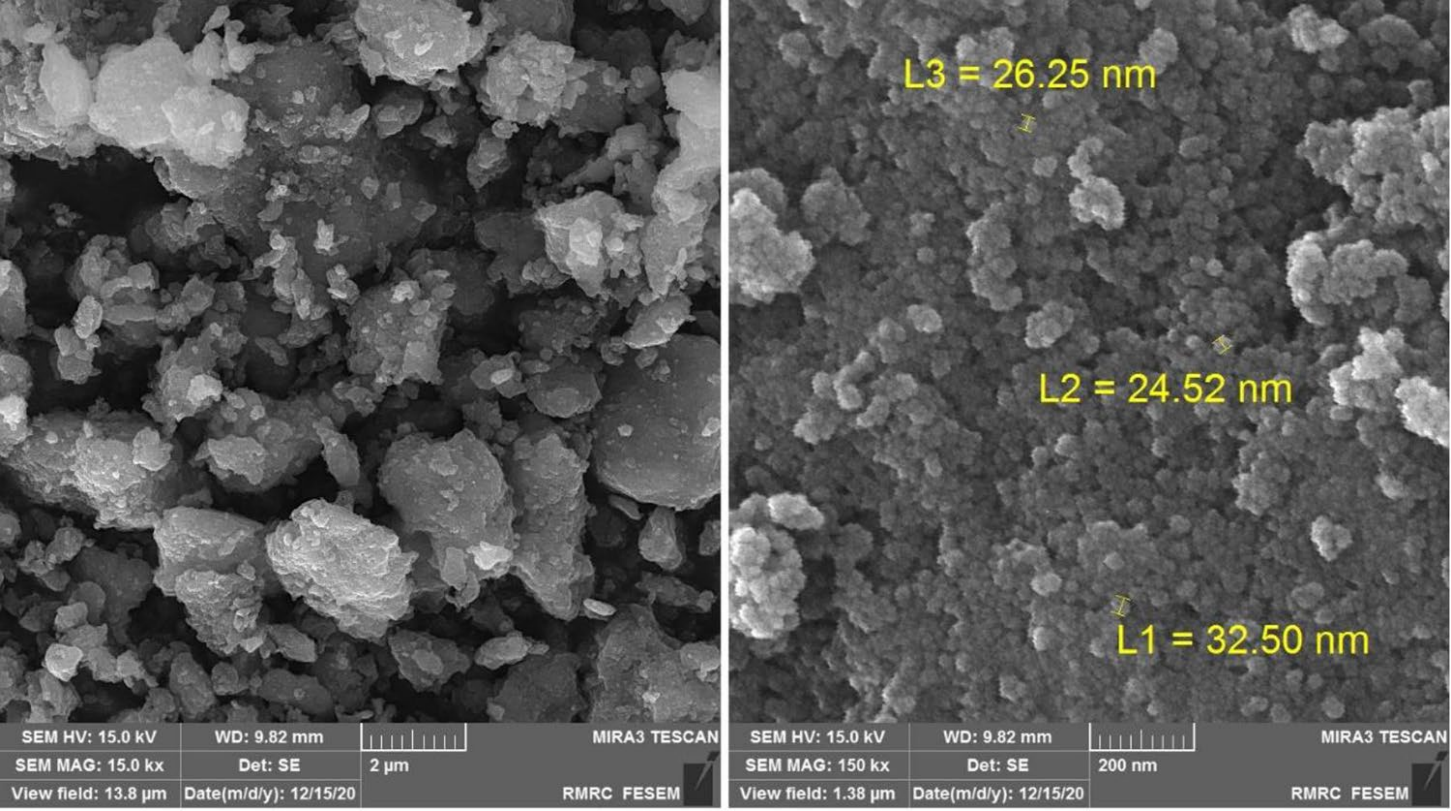
FESEM analysis of MCS@PhTet@Cu(II).

FESEM images of MCS@PhTet@Cu(II) are presented in Fig. 4. The results show that the catalyst is irregularly shaped with a mean particle size of about 24–33 nm.

**Figure 5:**
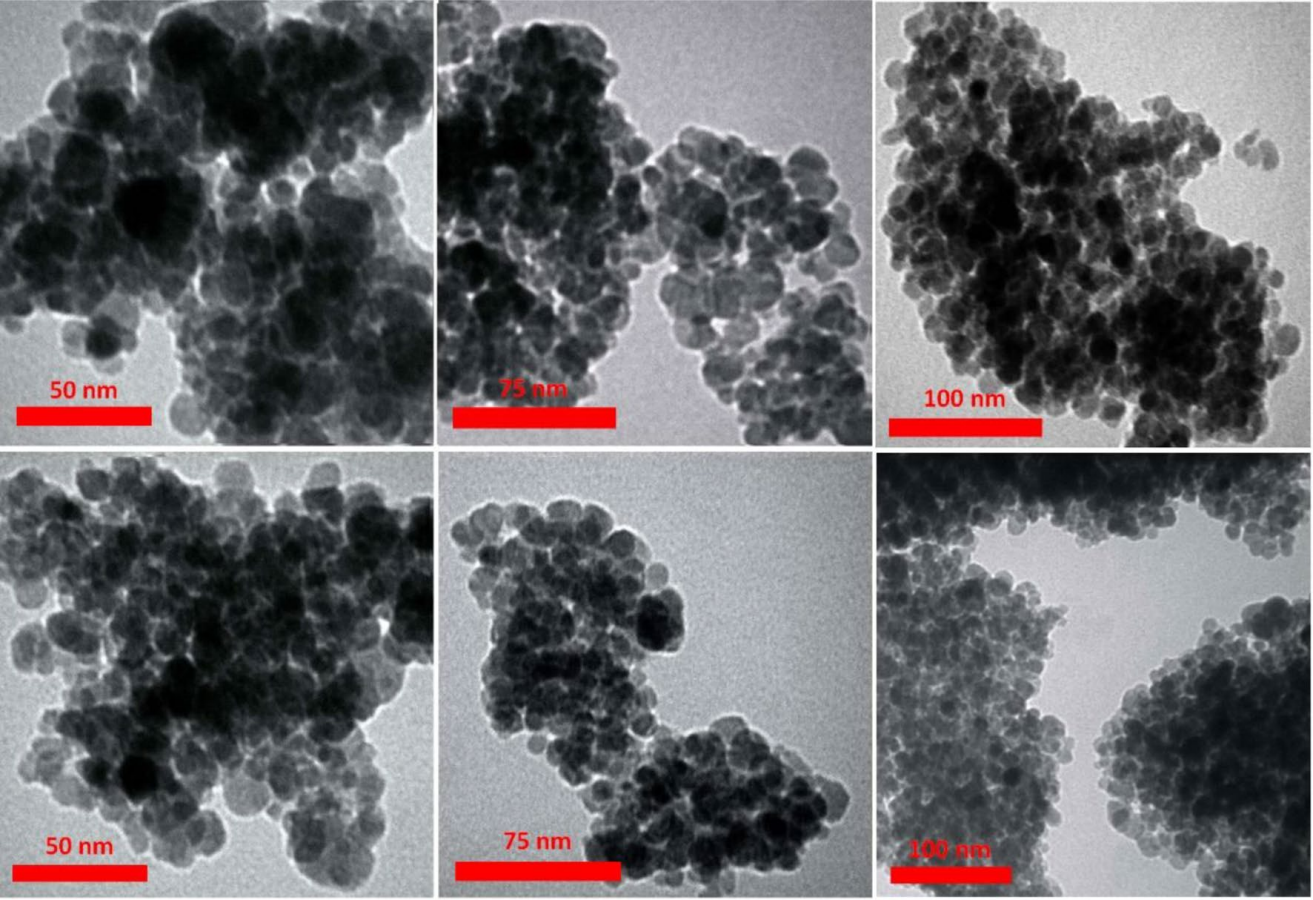
TEM analysis of MCS@PhTet@Cu(II).

According to the TEM analysis, the catalyst shows an average particle size of about 28 nm (Fig. 5). Moreover, the accumulation of iron oxide NPs is observed as dark spots.

**Figure 6:**
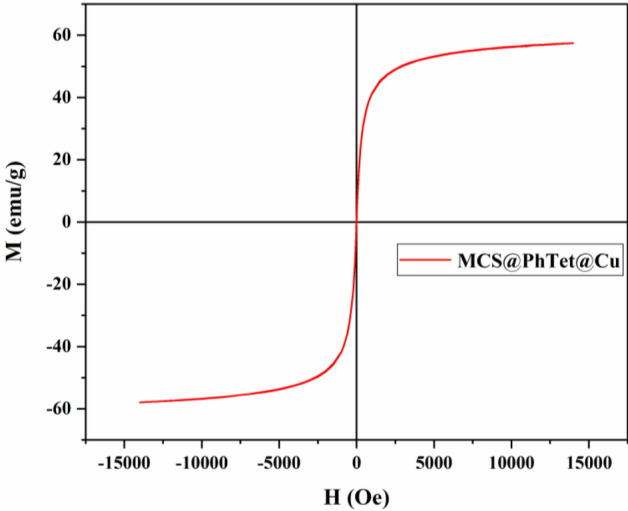
VSM spectrum of MCS@PhTet@Cu(II).

The magnetic behavior of the catalyst was examined by VSM analysis (Fig. 6). According to the results, MCS@PhTet@Cu(II) represents high magnetic sensitivity and is hence magnetically separable.

**Figure 7:**
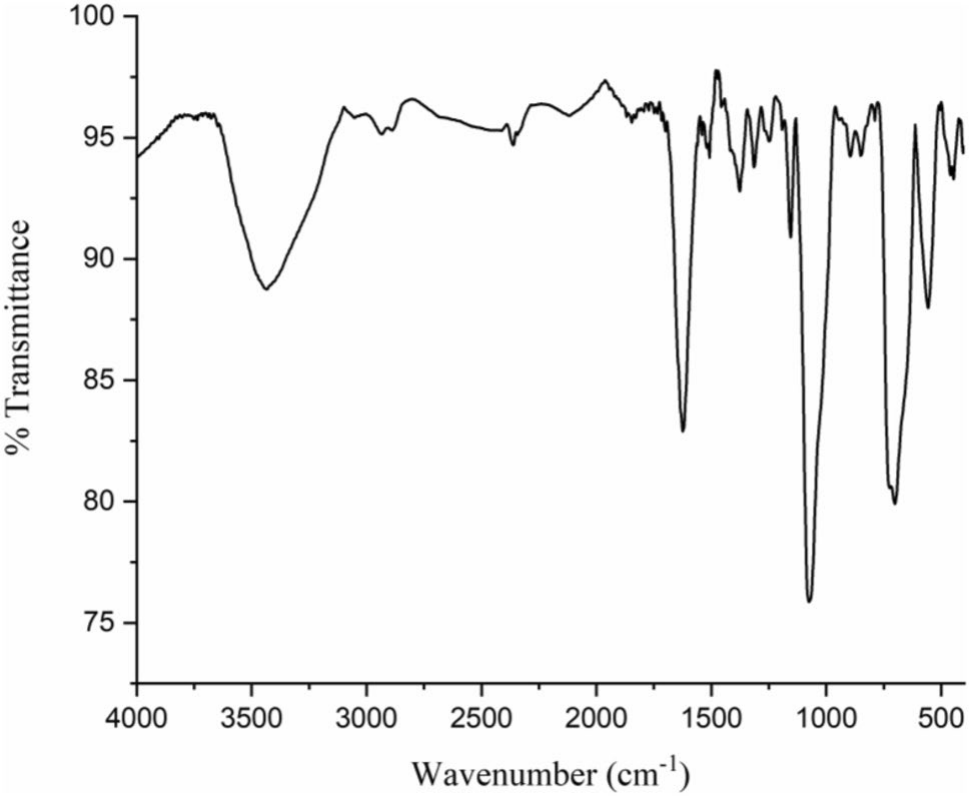
FT-IR spectrum of MCS@PhTet@Cu(II).

FT-IR analysis was also used to characterize MCS-PhTet-Cu(II) (Fig. 7). The peak at 557 cm^−1^ corresponds to the Fe–O, indicating the magnetic nanoparticles. In addition, the peak in the range of 3200–3500 cm^−1^ is correlated to NH and OH groups of chitosan and OH functional group in Fe_3_O_4_. In Fig. 7, the peaks at around 1153 cm^−1^ and the 1400–1670 cm^−1^ range correspond to C–O, N=N, C=N, and C=C stretching vibrations, respectively.

### Synthesis of 3-imino-2-phenylisoindolin-1-one derivatives

The catalytic performance of MCS@PhTet@Cu(II) catalyst was examined in the reaction between benzoyl chloride and *N*-(4-chlorophenyl)cyanamide by employing various quantities of the catalyst in EtOH at 25 °C under US irradiation (Table 1). According to the green principle; that is, high yields, eco-friendliness, and short reaction times, EtOH was chosen as the best solvent for this protocol. As observed in Table 1, the amount of the catalyst notably affects the reaction. The best result was obtained using 1 mmol of benzoyl chloride, 1 mmol of *N*-(4-chlorophenyl)cyanamide, and 30 mg of MCS@PhTet@Cu(II) (with 1.93 mol% Cu content) in 10 mL of EtOH solvent (Table 1, entry 2) and no more enhancement in the yield or reaction time was observed by additional catalyst (Entry 3). A decrease in catalyst loading reduced the yield of the product and the reaction became sluggish (Entry 1). To study the effect of US irradiation on the reaction yield, the reaction was performed under thermal conditions at 70 °C in the absence of ultrasound (entry 4). As a result, the reaction yield in the synthesis of 2-(4-cholorophenyl)-3-iminoisoindolin-1-one under US irradiation is greater than that under thermal conditions. The reaction was performed in various solvents such as DMF and DMSO at 25 °C under US irradiation (Table 1, entries 5 and 6). Table 1 shows that ethanol solvent gave better yield than DMF and DMSO solvents. Limiting the usage of EtOH solvent increases the green characteristics of the reaction and facilitates the workup/isolation of the product formed in high yield. Therefore, EtOH was found to be the optimal solvent.

**Table 1:** Synthesis of 2-(4-cholorophenyl)-3-iminoisoindolin-1-one using various quantities of the catalyst in the different solvents at 25 °C under US irradiation and thermal conditions.

Entry	Catalyst (mol% Cu content)	Solvent (mL)	Time (h)	Yield (%)^a^
1	1.29	EtOH (10)	6	62
2	1.93	EtOH (10)	5	84
3	2.58	EtOH (10)	5	84
4	1.93	EtOH (10)	5	66^b^
5	1.93	DMF (6)	5	70
6	1.93	DMSO (6)	5	68

Reaction conditions: Benzoyl chloride (1 mmol), *N*-(4-chlorophenyl)cyanamide (1 mmol), catalyst, solvent, room temperature, US irradiation.

^a^Isolated yield.

^b^The reaction was carried out at 70 °C in the absence of ultrasound.

With the optimized conditions in hand, the applicability of MCS@PhTet@Cu(II) was investigated for cycloaddition reaction of benzoyl chloride with different derivatives of arylcyanamides containing electron donating or withdrawing substituents (Table 2). It is obvious that our system is general and applicable to different substituted arylcyanamides. In addition, the electronic effects do not play much of a role in the product yields. In every case, the corresponding product was obtained in high yields. Furthermore, MCS@PhTet@Cu(II) can be easily removed using an external magnet and reused for several runs with no significant decay in the performance. These advantages make our method a valuable alternative for the reaction of benzoyl chloride with arylcyanamides.

**Table 2:** Synthesis of 3-imino-2-phenylisoindolin-1-one derivatives using various arylcyanamides in EtOH.

Entry	Arylcyanamide	Product	Yield (%)^a^
1			84
2			84
3			85
4			84
5			82
6			83
7			83

Reaction conditions: Benzoyl chloride (1 mmol), arylcyanamide (1 mmol), catalyst (1.93 mol% Cu content), EtOH (10 mL), room temperature, US irradiation, 5 h.

^a^Isolated yield.

**Figure 8:**
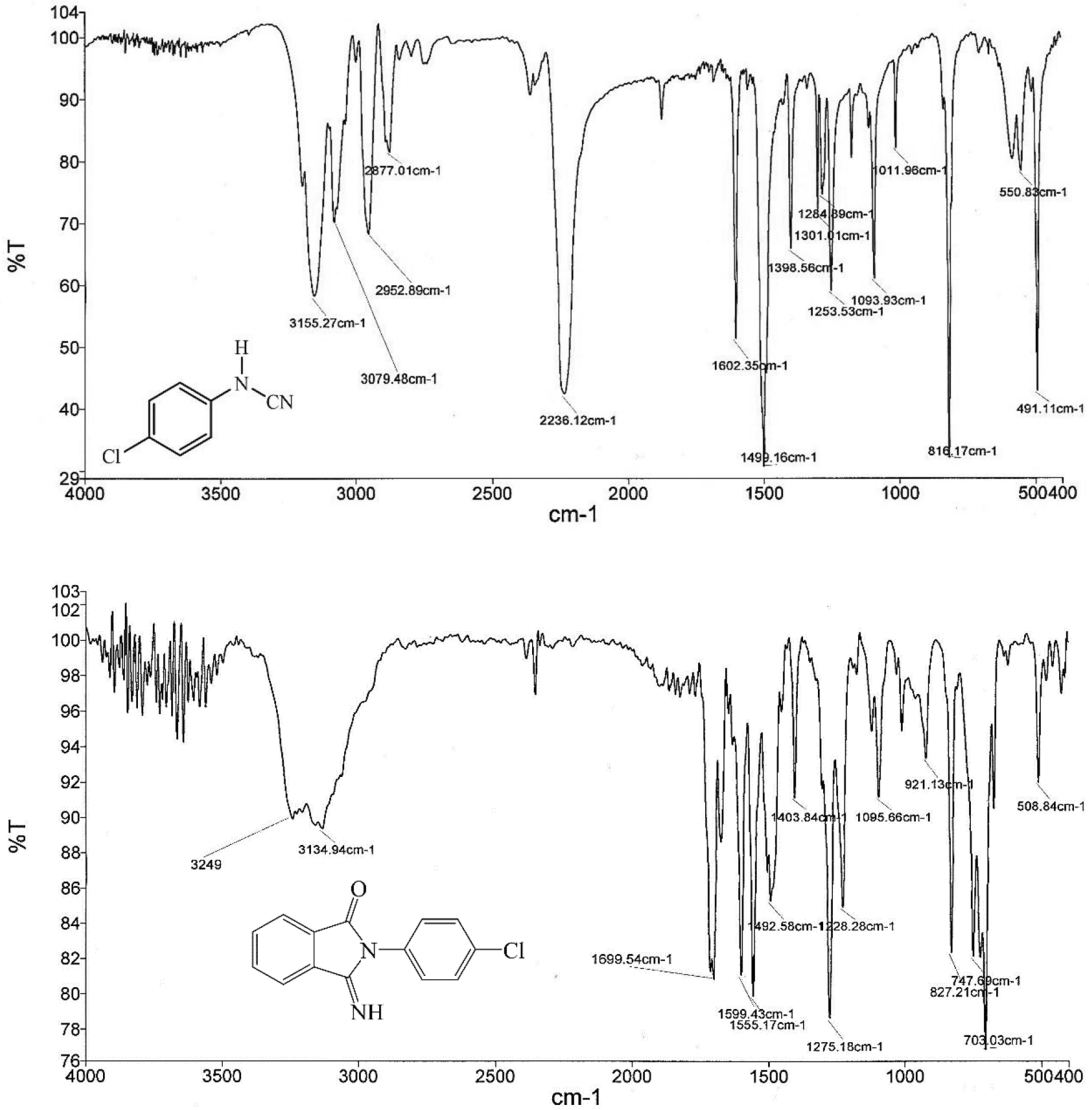
FT-IR spectra of *N*-(4-chlorophenyl)cyanamide (top) and 2-(4-chlorophenyl)-3-iminoisoindolin-1-one (bottom).

**Figure 9:**
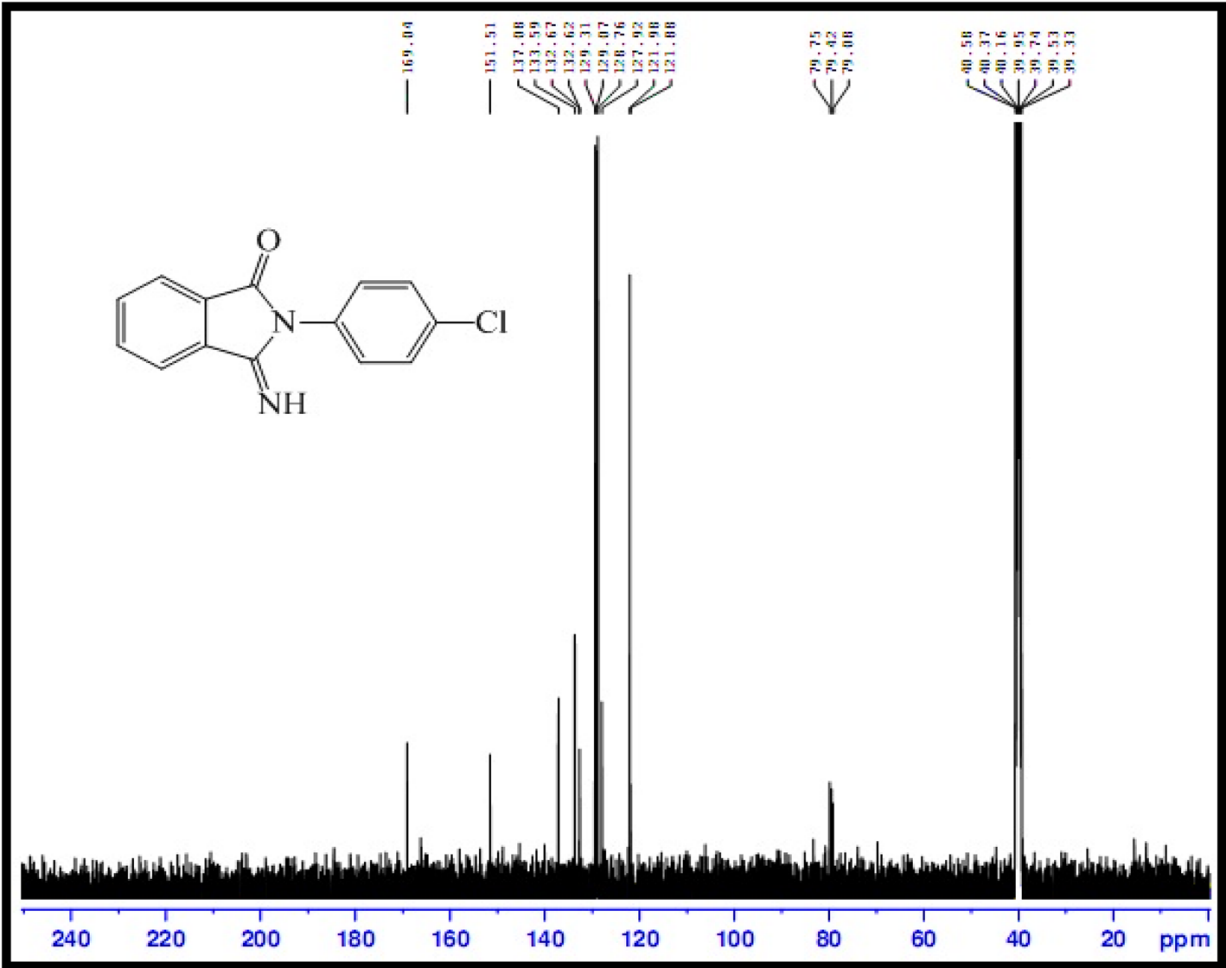
^13^CNMR spectrum of 2-(4-chlorophenyl)-3-iminoisoindolin-1-one.

At the end of the reaction, the products were purified and characterized by melting point and FT-IR and NMR analyses. Most of the obtained products are known compounds whose physical and spectral data match those of authentic samples^57^. The disappearance of the absorption band in 2200–2300 cm^−1^ range corresponding to the CN functional group of arylcyanamides and the appearance of the adsorption peaks related to the NH and C=O groups of products in the ranges of 3234–3438 and 1697–1717 cm^−1^, respectively, confirm the formation of 3-imino-2-phenylisoindolin-1-one derivatives (Fig. 8). In the ^13^CNMR spectrum, two peaks were observed corresponding to the imide and carbonyl carbon (Fig. 9).

### Predicted mechanism

**Scheme 3:**
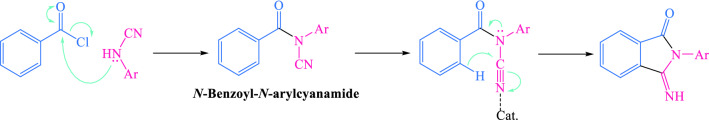
Predicted pathway for the synthesis of 3-imino-2-phenylisoindolin-1-one derivatives.

The predicted mechanistic pathway for the reaction between benzoyl chloride and arylcyanamides using MCS@PhTet@Cu(II) catalyst at room temperature under US irradiation to synthesize 3-imino-2-phenylisoindolin-1-ones is shown in Scheme 3^57^. The reaction proceeds by the activation of the cyanide group with copper catalyst, followed by intramolecular cycloaddition. The formation of the product is confirmed by melting point, FT-IR, and NMR analysis.

### Recyclability of MCS@PhTet@Cu(II)

**Figure 10:**
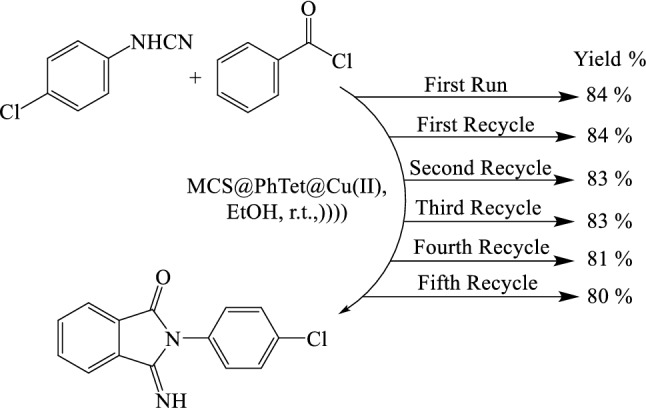
Reusability of MCS@PhTet@Cu(II) in the synthesis of 2-(4-cholorophenyl)-3-iminoisoindolin-1-one.

The recyclability of a catalyst is one of its promising features. The recyclability of MCS@PhTet@Cu(II) was investigated in a model reaction between benzoyl chloride and *N*-(4-chlorophenyl)cyanamide in EtOH at room temperature under US irradiation. Upon the completion of the reaction, the catalyst was removed from the reaction medium by an external magnet; the solvent was evaporated, and the solid residue was washed with water. The product was then purified by recrystallization using aqueous ethanol. The catalyst was reused in five consecutive runs with no noteworthy decrease in the catalytic performance (Fig. 10), which proves its high stability. The leaching phenomenon was measured by ICP-MS analysis after five run. The results of heterogeneity test confirm slight leaching of Cu species during the reaction and the heterogeneity of MCS@PhTet@Cu(II) catalyst. Less than 0.1% of the Cu was observed in the solution during the synthesis of 2-(4-cholorophenyl)-3-iminoisoindolin-1-one. These results confirm the structural stability of MCS@PhTet@Cu(II) and the strong coordinating of Cu(II) on MCS@PhTet.

## Experimental

### Materials and instruments

All the materials were obtained from the Aldrich and Merck Chemical Co. Several spectroscopic methods including FT-IR, ^1^H NMR, and ^13^C NMR, and melting points were employed to characterize the products. NMR analysis was carried out in DMSO and acetone solvents. ^1^H NMR and FT-IR (KBr) spectra were recorded on Bruker Avance DRX 400 MHz instrument and Perkin-Elmer 781 spectrophotometer, respectively. Ultrasonication was performed using a Digital Pro 2600 s ultrasound cleaner with a frequency and output power of 40 kHz and 180 W, respectively.

### Synthesis of magnetic chitosan

0.25 g of CS was dissolved in 1% (V/V) acetic acid solution (50 mL) to obtain the chitosan solution. 2 g of Fe_3_O_4_ NPs were then mixed with this solution and the mixture obtained was stirred at room temperature. After 30 min, 50 mL of NaOH (1 M) were slowly added. Lastly, the prepared Fe_3_O_4_-chitosan (MCS) was separated by an external magnet, washed consecutively with ethanol, water, and acetone and dried.

### Synthesis of magnetic chitosan@5-phenyl-1*H*-tetrazole

To synthesize magnetic chitosan@5-phenyl-1*H*-tetrazole (MCS@PhTet), firstly, 5 mmol of (3-chloropropyl)trimethoxysilane (TMOS) were added to 5 mmol of 5-phenyl-1*H*-tetrazole and the resulting mixture was refluxed in EtOH for 24 h (Solution A). In another flask, 1.5 g of MCS were dispersed in EtOH for 30 min by ultrasonic irradiation (Solution B). Then, 5 mmol of K_2_CO_3_ and solution A were slowly added to the dispersed chitosan solution (Solution B) and the mixture formed was stirred at 50 °C for 24 h.

### Preparation of MCS@PhTet@Cu(II)

**Scheme 4:**
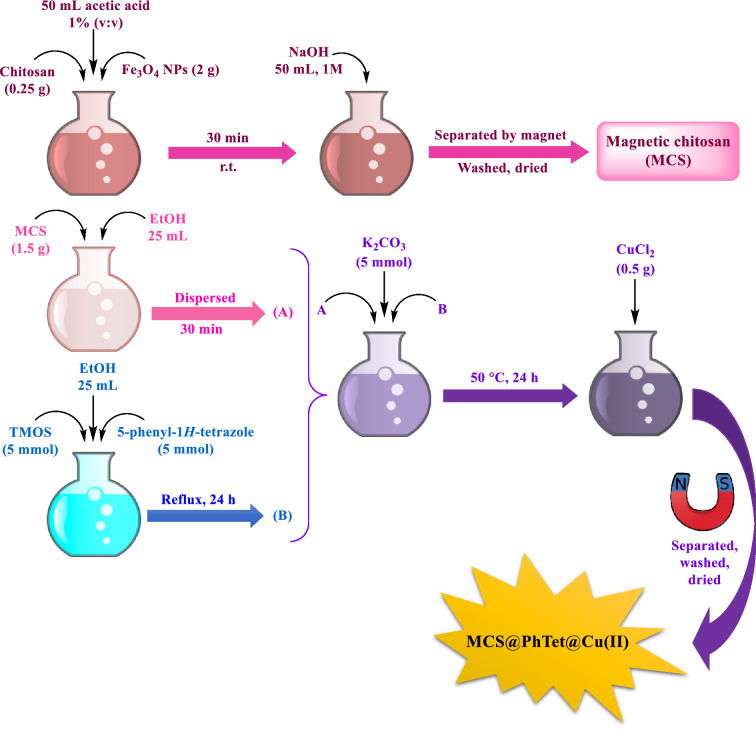
Synthesis of MCS@PhTet@Cu(II).

Finally, MCS@PhTet and 0.5 g of CuCl_2_ were mixed in refluxing EtOH for 24 h. The resulting complex was separated by an external magnet, washed with EtOH, and air dried. Scheme 4 represents the step-by-step preparation of MCS@PhTet@Cu(II).

### General method for the preparation of 3-imino-2-phenylisoindolin-1-one derivatives

A mixture of benzoyl chloride (1 mmol), arylcyanamide (1 mmol), and MCS@PhTet@Cu(II) catalyst (1.93 mol% Cu content) in EtOH (10 mL) was stirred at 25 °C under US irradiation. At the end of the reaction (monitored by TLC), the magnetic catalyst was separated by a magnet, the solvent was removed, and the solid precipitate was washed with water. The product was purified by recrystallization in aqueous ethanol. Most of the synthesized products described herein are known and their melting points were found to be consistent with previously reported values in the literature^57^.

### Characterization data of new product

#### 2-(2-Cholorophenyl)-3-iminoisoindolin-1-one (Table 2, entry 2)

M.p. 251–253 °C; ^1^H NMR (400 MHz, DMSO-*d*_*6*_) δ_H_ = 10.87 (s, 1H), 8.03 (d, *J* = 7.6 Hz, 2H), 7.69–7.63 (m, 3H), 7.56 (t, *J* = 7.6 Hz, 2H), 7.42 (d, *J* = 8.8 Hz, 2H); ^13^C NMR (100 MHz, DMSO-*d*_*6*_) δ_C_ = 169.0, 151.5, 137.1, 133.6, 132.67, 132.62, 129.3, 129.1, 128.7, 127.9, 121.9, 121.8; FT-IR (KBr, cm^−1^) 3438, 3246, 1701, 1598, 1556, 1489, 1402, 1274, 1227, 826, 702, 544.

## Conclusion

In this work, a novel approach has been developed for the preparation of MCS@PhTet@Cu(II) magnetically separable and heterogeneous catalyst using magnetic chitosan and 5-phenyl-1*H*-tetrazole as the support and ligand, respectively. MCS@PhTet@Cu(II), a Lewis acid catalyst, was prepared via the immobilization of (Cu(II)-5-phenyl-1*H*-tetrazole) copper complex on Fe_3_O_4_-chitosan and thoroughly characterized by TEM, FESEM, VSM, FT-IR, XRD, EDS, and ICP-MS techniques. The catalyst has been used for the preparation of 3-imino-2-phenylisoindolin-1-one derivatives by the reaction between benzoyl chloride and arylcyanamides in EtOH solvent at room temperature under US irradiation. The products were isolated without use of column chromatography. This procedure has many advantages such as the use of natural/inexpensive compounds, simple reaction set-up, mild reaction conditions, clean reaction profile, avoiding toxic and hazardous solvents, moderate reaction time, high yield, easy separation of the products without any tedious separation techniques, easy workup, avoidance of column chromatography, stability of the catalyst, and separation ability using an external magnet. The reusability study of the catalyst showed that the catalyst can be reused efficiently for up to five consecutive cycles.
